# The Sesquiterpene Synthase PtTPS5 Produces (1*S*,5*S*,7*R*,10*R*)-Guaia-4(15)-en-11-ol and (1*S*,7*R*,10*R*)-Guaia-4-en-11-ol in Oomycete-Infected Poplar Roots

**DOI:** 10.3390/molecules26030555

**Published:** 2021-01-21

**Authors:** Nathalie D. Lackus, Jennifer Morawetz, Houchao Xu, Jonathan Gershenzon, Jeroen S. Dickschat, Tobias G. Köllner

**Affiliations:** 1 Max Planck Institute for Chemical Ecology, Hans-Knöll-Strasse 8, 07745 Jena, Germany; nlackus@ice.mpg.de (N.D.L.); jenny.morawetz@gmail.com (J.M.); gershenzon@ice.mpg.de (J.G.); 2 Kekulé-Institute of Organic Chemistry and Biochemistry, University of Bonn, Gerhard-Domagk-Strasse 1, 53121 Bonn, Germany; houchao.xu@uni-bonn.de (H.X.); dickschat@uni-bonn.de (J.S.D.)

**Keywords:** sesquiterpene synthase, *Populus trichocarpa*, oomycete, *Phytophthora cactorum*, plant defense

## Abstract

Pathogen infection often leads to the enhanced formation of specialized plant metabolites that act as defensive barriers against microbial attackers. In this study, we investigated the formation of potential defense compounds in roots of the Western balsam poplar (*Populus trichocarpa*) upon infection with the generalist root pathogen *Phytophthora cactorum* (Oomycetes). *P. cactorum* infection led to an induced accumulation of terpenes, aromatic compounds, and fatty acids in poplar roots. Transcriptome analysis of uninfected and *P. cactorum*-infected roots revealed a terpene synthase gene *PtTPS5* that was significantly induced upon pathogen infection. PtTPS5 had been previously reported as a sesquiterpene synthase producing two unidentified sesquiterpene alcohols as major products and hedycaryol as a minor product. Using heterologous expression in *Escherichia coli*, enzyme assays with deuterium-labeled substrates, and NMR analysis of reaction products, we could identify the major PtTPS5 products as (1*S*,5*S*,7*R*,10*R*)-guaia-4(15)-en-11-ol and (1*S*,7*R*,10*R*)-guaia-4-en-11-ol, with the former being a novel compound. The transcript accumulation of *PtTPS5* in uninfected and *P. cactorum*-infected poplar roots matched the accumulation of (1*S*,5*S*,7*R*,10*R*)-guaia-4(15)-en-11-ol, (1*S*,7*R*,10*R*)-guaia-4-en-11-ol, and hedycaryol in this tissue, suggesting that PtTPS5 likely contributes to the pathogen-induced formation of these compounds in planta.

## 1. Introduction

Plants are constantly under attack from a multitude of pests, including pathogens and herbivores. Such biotic stresses often induce the formation of specialized plant metabolites that play major roles in plant defense. Terpenoids represent the largest class of natural compounds, and to date, more than 200,000 terpenoids are known, of which ~40,000 can be produced by plants [1]. Beside a few roles in primary metabolism and physiology, most plant terpenes function as specialized metabolites in processes such as plant signaling and defense. Volatile mono- and sesquiterpenes, for example, have been described as repellants for herbivores or attractants for beneficial insects and animals e.g., [2,3,4]. Non-volatile terpenoids, however, can act as phytoalexins and protect the plant against pathogen infection by inhibiting the growth and/or development of the attacking pathogen [5]. The sesquiterpene-derived zealexins and the diterpene-derived kauralexins in the grasses are well known examples for antimicrobial and locally accumulating plant terpenoids that are produced in response to pathogen attack [6,7].

The biosynthesis of terpenes starts with the formation of isopentenyl diphosphate (IPP) and dimethylallyl diphosphate (DMAPP), which represent the C5 building blocks for all terpenes. IPP and DMAPP can be condensed by isopentenyl diphosphate synthases (IDS) to form a variety of prenyl diphosphates with various chain lengths, including geranyl diphosphate (GPP, C_10_), (*E,E*)-farnesyl diphosphate (FPP, C_15_), and (*E,E,E*)-geranylgeranyl diphosphate (GGPP, C_20_). The prenyl diphosphates are substrates for terpene synthases (TPS), which catalyze the formation of the basic mono-(C_10_), sesqui-(C_15_), and diterpene (C_20_) skeletons [8]. The terpenes formed can be stored in the plant tissue or released as volatiles. Additionally, terpenes can act as substrates for modifying enzymes such as cytochrome P450 monooxygenases, *O*-methyltransferases, and acyltransferases [8,9,10].

In recent years, we investigated the formation of defense terpenes in the model tree species Western balsam poplar (*Populus trichocarpa*). Nineteen out of the 38 *TPS* genes found in the *P. trichocarpa* genome and three *IDS* genes involved in GPP and FPP formation have been cloned and characterized so far [11,12,13,14,15]. Most of these genes are significantly upregulated upon leaf or root herbivory, indicating that their terpene products are involved in plant defense against insect herbivores. However, whether poplar terpenes can also be formed as potential phytoalexins in response to pathogen attack is unclear. The aim of this study was to investigate the formation of defense compounds including terpenes in *P. trichocarpa* roots upon infection with a plant pathogen. The root rot-causing hemibiotrophic generalist oomycete *Phytophthora cactorum* was selected as a model organism because of its broad host specificity and economic importance. It can infect more than 200 plant species, including important crops such as apple trees and strawberries or ornamentals such as orchids. Transcriptome sequencing and RT-qPCR analysis revealed a sesquiterpene synthase gene *PtTPS5*, which was highly expressed in *P. cactorum*-infected roots but not in non-infected control roots. Enzyme assays with recombinant PtTPS5 and (*E,E*)-FPP as substrate and subsequent NMR analysis of TPS reaction products allowed the identification of two sesquiterpene alcohols that also accumulated in infected poplar roots. We propose that the PtTPS5 sesquiterpenes or their potential conversion products function as a defensive barrier against pathogen infection in poplar roots.

## 2. Results

### 2.1. P. cactorum Infection Induces the Accumulation of Terpenes, Aromatic Compounds, and Fatty Acids in P. trichocarpa Roots

To investigate the formation of potential defense compounds upon pathogen infection in poplar roots, young *P. trichocarpa* trees were grown in liquid medium and inoculated with a zoospore suspension of the generalist oomycete *P. cactorum*. Roots were harvested five days after inoculation, extracted with hexane and the extracts were analyzed using gas chromatography-mass spectrometry (GC-MS). Beside traces of the monoterpenes limonene and 1,8-cineole, the monoterpene alcohol α-terpineol, the sesquiterpene alcohol elemol and two so far unidentified sesquiterpene alcohols were detected. Elemol most likely represents a rearrangement product of hedycaryol formed during GC-MS analysis. In general, germacrane sesquiterpenoids such as hedycaryol or germacrene A are well known to undergo thermal Cope rearrangements to elemol or β-elemene, respectively [16]. Thus, the thermal formation of elemol from hedycaryol under the conditions of the GC analysis is more likely than a direct enzymatic formation, which has never been described and would be difficult to understand mechanistically. The two unidentified sesquiterpene alcohols were later identified in this study as (1*S*,5*S*,7*R*,10*R*)-guaia-4(15)-en-11-ol and (1*S*,7*R*,10*R*)-guaia-4-en-11-ol (see Section 2.3). While limonene and 1,8-cineole could not be quantified due to low amounts and partial overlap with other peaks, α-terpineol, elemol, and the two unidentified sesquiterpene alcohols showed a significantly higher accumulation in *P. cactorum*-infected roots compared to uninfected control roots (Table 1, Supplemental Figure S1). *P. cactorum* mycelium grown in liquid poplar growth medium in the absence of poplar roots showed no terpene accumulation (Supplemental Figure S2), suggesting that the terpenes detected in *P. cactorum*-infected roots were produced by the plant and not the oomycete.

In addition to the terpenes, a number of aromatic compounds including benzylalcohol, salicylaldehyde, 2-phenylethanol, and benzyl salicylate, some fatty acids, and the fatty acid aldehyde myristaldehyde could be detected in the root hexane extracts (Table 1). Two of the aromatic compounds namely benzylalcohol and 2-phenylethanol, almost all fatty acids, and myristaldehyde were significantly upregulated upon oomycete infection. With the exception of myristic acid, all fatty acids also occurred in hexane extracts made from *P. cactorum* mycelium grown in the absence of poplar roots (Supplemental Figure S2).

Salicinoids, a group of salicylalcohol-derived glucosides, are major defense compounds in the Salicaceae (reviewed in Böckler et al. [17]). To test whether salicinoid levels were influenced by the *P. cactorum* treatment, root material was extracted with methanol and the extracts were analyzed using high performance liquid chromatography (HPLC)-UV and liquid chromatography-tandem mass spectrometry (LC-MS/MS). While the accumulation of most of the measured salicinoids including salicin, salirepin, salirepin-7-sulfate, salicortin, tremulacin, and homaloside D was not influenced by the oomycete treatment, salicin-7-sulfate showed a small but significant induction upon pathogen infection (Table 2).

Infection of *P. trichocarpa* roots by *P. cactorum* was verified by measuring the transcript accumulation of the *Phytophthora*-specific Ras-related protein *Ypt1* [18] in the root material using RT-qPCR. *Ypt1* transcripts could be detected in *P. cactorum*-infected roots but not in uninfected control roots (Supplemental Figure S1), indicating a successful infection of the plant.

### 2.2. Transcriptome Analysis of Infected and Non-Infected Poplar Roots Revealed a Sesquiterpene Synthase Gene PtTPS5 that Is highly Induced upon P. cactorum Infection

**Figure 1:**
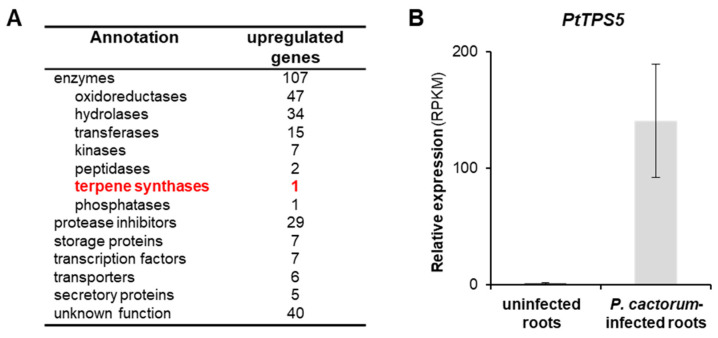
Transcript accumulation of the sesquiterpene synthase gene *PtTPS5* is upregulated after *Phytophthora cactorum* infection in *Populus trichocarpa* roots. (**A**) RNAseq, subsequent read mapping, and EDGE (estimated degree of gene expression) analysis was performed to identify genes significantly higher expressed in *P. cactorum*-infected roots compared to uninfected control roots. Genes with a fold change > 5 (false discovery rate < 0.01%; *n* = 4) were considered as upregulated. (**B**) Relative gene expression of *PtTPS5.* Means and SE of RPKM values are shown (*n* = 4). EDGE test (*p* = 4.9 × 10^−19^, weighted difference = 0.000254923).

In order to identify genes involved in the *P. cactorum*-induced plant defense response, especially in terpene formation, we sequenced and analyzed the transcriptomes of infected and non-infected *P. trichocarpa* roots. Mapping the sequence reads onto the *P. trichocarpa* gene set revealed 201 genes that were significantly upregulated (fold change > 5) upon *P. cactorum* infection (Figure 1A, Supplemental Table S1). Among these genes, 107 encoded enzymes, including a highly upregulated terpene synthase (PtTPS5, Potri.005g095500). PtTPS5 has recently been reported as sesquiterpene synthase producing unidentified sesquiterpene alcohols as major products and hedycaryol as a minor product [12]. Notably, the relatively high RPKM values (average ~140) for *PtTPS5* in *P. cactorum*-infected roots were comparable to those of a variety of protease inhibitor genes known to be involved in plant defense (Figure 1B, Supplemental Table S1).

### 2.3. PtTPS5 Produces (1S,5S,7R,10R)-guaia-4(15)-en-11-ol and (1S,7R,10R)-guaia-4-en-11-ol as Major Products

**Figure 2:**
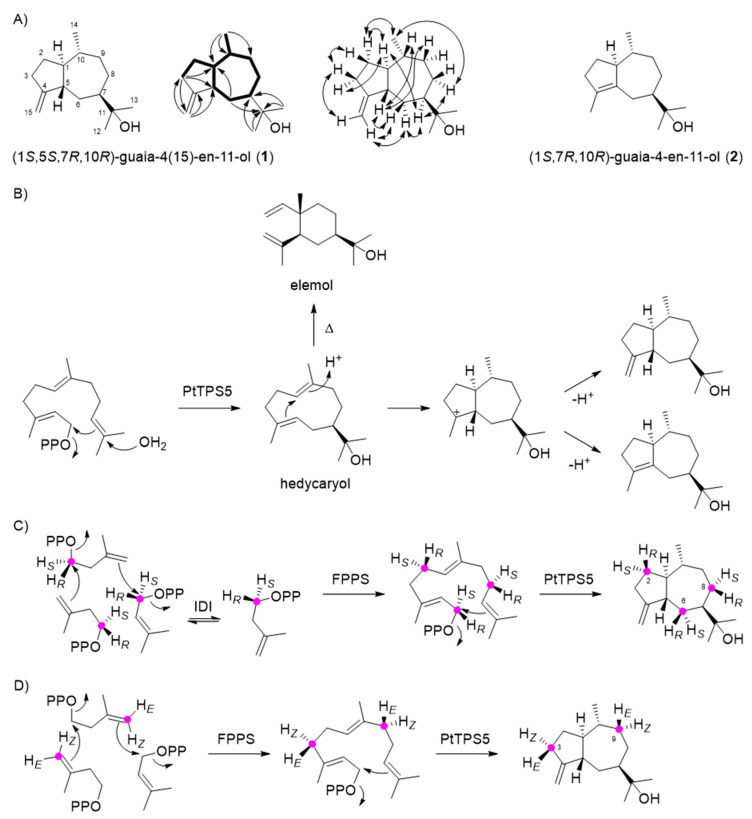
PtTPS5 produces (1*S*,5*S*,7*R*,10*R*)-guaia-4(15)-en-11-ol (**1**), (1*S*,7*R*,10*R*)-guaia-4-en-11-ol (**2**), and minor amounts of hedycaryol *in vitro*. (**A**) Structure elucidation (bold lines: H,H-COSY correlations, single-headed arrows: HMBC correlations, double-headed arrows: NOESY correlations). Carbon numbering is not systematic, but follows the FPP numbering. (**B**) Biosynthetic model for the cyclisation from FPP to the sesquiterpene alcohols and Cope rearrangement of hedycaryol to elemol under the thermal conditions of GC-MS analysis. Determination of the absolute configuration of (1*S*,5*S*,7*R*,10*R*)-guaia-4(15)-en-11-ol by enantioselective deuteration using (**C**) (*R*)- or (*S*)-(1-^13^C,1-^2^H)IPP with IDI, FPPS and PtTPS5, and (**D**) (*E*)- or (*Z*)-(4-^13^C,4-^2^H)IPP with FPPS and PtTPS5.

To elucidate the structure of the unidentified PtTPS5 products, the gene was synthesized, cloned into the bacterial expression vector pET100/D-TOPO, and heterologously expressed in *Escherichia coli*. Purified recombinant protein was incubated with (*E,E*)-FPP as substrate. GC-MS analysis of the products revealed the formation of hedycaryol, detected as its Cope rearrangement product elemol, and two coeluting sesquiterpene alcohols that were purified by column chromatography, followed by structure elucidation through one- and two-dimensional NMR spectroscopy (Table 3), resulting in the structures of the new compound guaia-4(15)-en-11-ol (**1**) and the known guaia-4-en-11-ol (**2**) that was previously reported from *Bulnesia sarmientoi* (Figure 2A) [19]. A biosynthetic hypothesis for these sesquiterpene alcohols suggests formation proceeds by 1,10-cyclisation and capture with water to form hedycaryol (Figure 2B). Its reprotonation at C10 can initiate a second cyclisation, as frequently observed for germacrane-type sesquiterpenes [20], to give a guaiane skeleton, followed by deprotonations from C15 or C5 to yield guaia-4(15)-en-11-ol and guaia-4-en-11-ol, respectively. The absolute configuration of guaia-4(15)-en-11-ol was determined through chemical correlation using stereoselectively deuterated precursors. The enzymatic conversion of (*R*)- or (*S*)-(1-^13^C,1-^2^H)IPP [21] with isopentenyl diphosphate isomerase from *E. coli* [21], FPP synthase from *Streptomyces coelicolor* [22], and PtTPS5 resulted in an enantioselective deuteration at C2, C6, and C8 of guaia-4(15)-en-11-ol with known configuration (Figure 2C), because it is well known that prenyl diphosphates are elongated by IPP with inversion of configuration at C1 [23]. The additional ^13^C-label allowed for a highly sensitive detection of HSQC signals for the bound hydrogens, while signals for the hydrogens substituted by deuterium vanished (Supplemental Figure S3A–C). The labelled carbons together with a full assignment of hydrogen signals by NOESY helped determine the relative orientation of the naturally present stereogenic centres in **1** and thus its absolute configuration as (1*S*,5*S*,7*R*,10*R*)-guaia-4(15)-en-11-ol. A second set of experiments was performed with DMAPP and (*E*)- or (*Z*)-(4-^13^C,4-^2^H)IPP [24] (Figure 2D), known to react with attack at C4 of IPP from the *Si* face under FPPS catalysis [23]. Further conversion by PtTPS5 resulted in the introduction of additional stereogenic probes at C3 and C9, and HSQC analysis gave consistent results regarding the absolute configuration of (1*S*,5*S*,7*R*,10*R*)-guaia-4(15)-en-11-ol (Supplemental Figure S3D–F). The absolute configurations of hedycaryol and (1*S*,7*R*,10*R*)-guaia-4-en-11-ol were assigned based on biosynthetic considerations (Figure 2B).

### 2.4. The Accumulation of (1S,5S,7R,10R)-guaia-4(15)-en-11-ol, (1S,7R,10R)-guaia-4-en-11-ol, and Hedycaryol in P. cactorum-Infected and Non-Infected Roots Matches the Expression of PtTPS5

**Figure 3:**
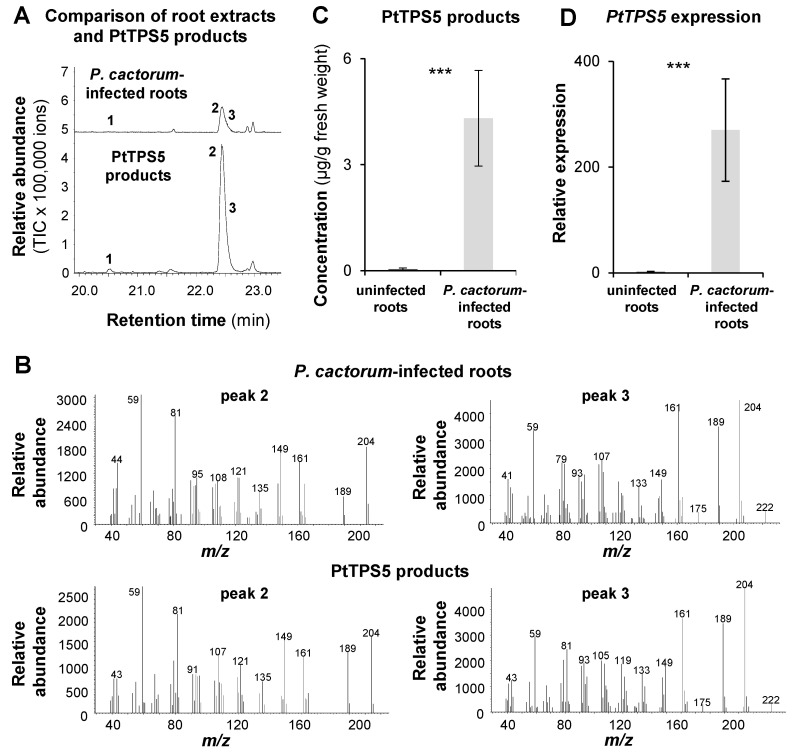
Accumulation of (1*S*,5*S*,7*R*,10*R*)-guaia-4(15)-en-11-ol and (1*S*,7*R*,10*R*)-guaia-4-en-11-ol and gene expression of poplar *PtTPS5* in uninfected and *Phytophtora cactorum*-infected *Populus trichocarpa* roots. (**A**) Terpenes were extracted with hexane from pulverized root material or from an assay containing recombinant PtTPS5 and (*E,E*)-FPP and analyzed using GC-MS. 1, elemol; 2, (1*S*,5*S*,7*R*,10*R*)-guaia-4(15)-en-11-ol; 3, (1*S*,7*R*,10*R*)-guaia-4-en-11-ol. (**B**) Mass spectra of (1*S*,5*S*,7*R*,10*R*)-guaia-4(15)-en-11-ol and (1*S*,7*R*,10*R*)-guaia-4-en-11-ol found in *P. cactorum*-infected roots and in enzyme assays of PtTPS5 (peaks 2 and 3). (**C**) Accumulation of PtTPS5 products in *P. trichocarpa* roots. Means and SE are shown (*n* = 7–8). Wilcoxon rank sum test (T = 36.00, *p* < 0.001). (**D**) Relative expression of *PtTPS5* determined by RT-qPCR. Means and SE are shown (*n* = 7–8). Student’s *t*-test (t = 7.626, *p* < 0.001). TIC = total ion count; ***, *p* ≤ 0.001.

To figure out whether the two unidentified sesquiterpene alcohols detected in *P. cactorum*-infected poplar roots were identical to the PtTPS5 products (1*S*,5*S*,7*R*,10*R*)-guaia-4(15)-en-11-ol and (1*S*,7*R*,10*R*)-guaia-4-en-11-ol, we analyzed and compared hexane extracts prepared from a PtTPS5 enzyme assay and oomycete-infected root material using GC-MS. Although the two sesquiterpene alcohols could not be separated completely under the GC conditions we used in this experiment, the peaks of the PtTPS5 products and the two unidentified alcohols in the root extract had identical retention times and highly similar mass spectra (Figure 3A,B). Notably, the minor PtTPS5 product hedycaryol could also be detected as trace compound in the root extract. *PtTPS5* gene expression in uninfected and *P. cactorum*-infected *P. trichocarpa* roots measured by RT-qPCR showed an expression pattern nearly identical to the accumulation pattern of the PtTPS5 products measured in the same tissue (Figure 3C,D). This indicates that PtTPS5 likely produces (1*S*,5*S*,7*R*,10*R*)-guaia-4(15)-en-11-ol, (1*S*,7*R*,10*R*)-guaia-4-en-11-ol, and traces of hedycaryol in *P. cactorum*-infected *P. trichocarpa* roots.

## 3. Discussion

In this study, we showed that infection of poplar roots by the generalist oomycete *P. cactorum* resulted in the induced accumulation of a number of potential defense compounds including terpenoids, aromatic compounds and fatty acids. Two of these compounds were exclusively produced in infected roots and could be identified as (1*S*,5*S*,7*R*,10*R*)-guaia-4(15)-en-11-ol and (1*S*,7*R*,10*R*)-guaia-4-en-11-ol, with the first one being a novel sesquiterpenoid (Table 1; Figure 2 and Figure 3). A recently reported terpene synthase, PtTPS5 [12], was found to form both sesquiterpene alcohols as major products and minor amounts of hedycaryol in vitro (Figure 3). Since *P. trichocarpa* possesses no other terpene synthase with high similarity to PtTPS5 [12], and *PtTPS5* is the only *TPS* gene significantly induced upon *P. cactorum* infection in roots (Supplemental Table S1), we conclude that the pathogen-induced accumulation of (1*S*,5*S*,7*R*,10*R*)-guaia-4(15)-en-11-ol, (1*S*,7*R*,10*R*)-guaia-4-en-11-ol, and hedycaryol is likely due to PtTPS5 activity *in vivo*. Infection of strawberry (*Fragaria vesca*) roots with *P. cactorum* has been shown to induce massive changes in the transcriptome, including the upregulation of the complete mevalonate pathway, two FPP synthase genes, and four putative sesquiterpene synthase genes with similarity to germacene D synthase [25]. Moreover, Yadav and colleagues reported that the infection of *Medicago truncatula* roots with the oomycete *Aphanomycus euteiches* led also to the expression of a sesquiterpene synthase gene [26]. The encoded enzyme MtTPS10 was shown to produce a blend of sesquiterpenes with the alcohol himachalol as the major component. Down regulation of *MtTPS10* resulted in increased susceptibility to the oomycete and a mixture of isolated MtTPS10 products inhibited mycelial growth and *A. euteiches* zoospore germination. However, since himachalol could not be detected in *A. euteiches*-infected roots, MtTPS10 alcohols are likely converted to other terpenoids as speculated by the authors [26]. Indeed, conversion of sesquiterpenes into polar compounds such as aldehydes and acids upon pathogen infection has been described in a number of plants. Pathogen-infected maize, for example, produces the sesquiterpene hydrocarbon β-macrocarpene, which is further converted to antimicrobial sesquiterpene acids called zealexins [7,27]. Kauralexins, another group of terpene acid phytoalexins found in maize, are produced from the diterpene *ent*-kaurene [6], and the sesquiterpene δ-cadinene acts as precursor for the formation of gossypol and other sesquiterpenoid phytoalexins in cotton [28,29]. In contrast to himachalol in infected *Medicago* roots, (1*S*,5*S*,7*R*,10*R*)-guaia-4(15)-en-11-ol and (1*S*,7*R*,10*R*)-guaia-4-en-11-ol accumulated in oomycete-infected poplar roots and thus could function as defense compounds themselves. However, considering the findings from the other plant systems described above, it is tempting to speculate that they might also be converted to other so far unknown antimicrobial defense compounds. Metabolism of terpenes often involves diverse hydroxylation and oxidation steps. Such reactions are in general catalyzed by cytochrome P450 monooxygenases or dioxygenases [10,30,31]. Our poplar transcriptome analysis revealed a number of putative P450 and dioxygenase genes that were strongly upregulated upon *P. cactorum* infection (Supplemental Table S1). Testing their enzymatic activity with PtTPS5 products as substrate will be a worthwhile aim for further studies.

Free fatty acids have been described to be involved in plant defense against various pathogens and herbivores [32,33,34]. They often act as signaling compounds or as precursors for signaling compounds [35], but can also directly impair the attacker [34]. Beside the two sesquiterpene alcohols (1*S*,5*S*,7*R*,10*R*)-guaia-4(15)-en-11-ol and (1*S*,7*R*,10*R*)-guaia-4-en-11-ol, we identified a number of fatty acids including myristic acid, pentadecanoic acid, palmitic acid, oleic acid, and stearic acid, that accumulated in substantial amounts in *P. cactorum*-infected roots (Table 1). Since pentadecanoic acid, palmitic acid, oleic acid, and stearic acid could also be detected in hexane extracts made from *P. cactorum* mycelium grown in the absence of roots (Supplemental Figure S2), it is likely that their increased accumulation in infected poplar roots is mainly caused by the oomycete itself. However, myristic acid was not found in *P. cactorum* mycelium and is produced by the poplar roots. Myristic acid has been shown to possess antimicrobial activity against diverse pathogenic fungi [34] and might act as a defense against the oomycete *P. cactorum*. Moreover, the related myristaldehyde has been reported as main constituent of many antimicrobial essential oils [36] and its oomycete-induced upregulation also indicates a function in poplar defense against pathogens.

## 4. Materials and Methods

### 4.1. Biological Material

Western balsam poplar (*Populus trichocarpa*, clone Muhle-Larsen, P&P Baumschule, Eitelborn, Germany) trees were propagated from monoclonal stem cuttings and grown under summer conditions in the greenhouse (24 °C, 60% rel. humidity, 16 h/8 h light/dark cycle) in hydroculture medium until they reached about 0.15 m in height. The hydroculture medium contained 7.05 g NaNO_3_, 3.05 g Ferty Basis 1 (Planta Düngemittel GmbH, Regenstauf, Germany), 1.36 g MgSO_4_, 0.04 g FeSO_4_·× 7H_2_O, and 0.05 g Titriplex^®^ V in a total volume of 5 L H_2_O.

*Phytophthora cactorum* (Oomycetes) was obtained from the Leibniz Institute DSMZ-German Collection of Microorganisms and Cell Cultures GmbH (Braunschweig, Germany). The generalist root pathogen was grown and sub-cultured via mycelial inoculation in petri dishes containing tomato juice medium. A 1.5 L quantity of medium contained 300 mL tomato juice (“Bio” quality from Netto supermarket), 4.5 g CaCO_3_ (Roth, Karlsruhe, Germany), and 11.25 g agar-agar, filled to full volume with triple distilled water (adjusted to pH 7.2) at room temperature.

### 4.2. Phytophthora Cactorum Treatment

Prior to the onset of the experiment, *P. cactorum* was freshly sub-cultured from mycelium and incubated in the dark at 25 °C. After seven days, plates were washed with ddH_2_O and the suspension obtained contained the *P. cactorum* sporangia. The number of sporangia was determined with a counting chamber and adjusted to a concentration of 3.78 × 10^5^ sporangia per 50 mL poplar hydroculture medium. The sporangia solution was stored for 30 min at 4 °C to induce the release of the zoospores. Each poplar tree was either placed in clean 50 mL poplar hydroculture medium (control; *n* = 7) or in 50 mL poplar hydroculture medium containing the above determined amount of *P. cactorum* sporangia (*P. cactorum*-infected; *n* = 8). Poplar trees were further grown for five days under summer conditions as described above (Section 4.1). Poplar hydroculture medium (50 mL) containing the same amount of *P. cactorum* sporangia (*P. cactorum* mycelium; *n* = 4) was cultivated for five days as described for the poplar trees. After five days of inoculation, poplar root material (average root weight of 0.41 g ± 0.05 (control) and 0.38 g ± 0.06 (*P. cactorum*-infected)) was harvested, immediately flash-frozen in liquid nitrogen, and stored at −80 °C until further processing. The *P. cactorum* mycelium samples were centrifuged at 15,000× *g* for 5 min, and the supernatant removed. The remaining mycelium was flash frozen in liquid nitrogen, and stored at −80 °C until further processing.

### 4.3. Hexane Extraction of Root Tissue and GC-MS/GC-FID Analysis

To determine the accumulation of non-polar compounds in poplar roots, 100 mg of ground root powder was extracted in a GC glass vial with 400 μL hexane including 10 ng/μL nonyl acetate as an internal standard. The extracts were shaken for one hour at 900 rpm and incubated overnight at room temperature. After centrifugation for 10 min at 5000× *g*, the supernatant was taken and subsequently analyzed via gas chromatography-mass spectrometry (GC-MS) and gas chromatography-flame ionization detection (GC-FID). The extraction of non-polar compounds from *P. cactorum* mycelium was performed as described above for the root tissue, except that 50 mg of the mycelium and 200 μL hexane were used.

Qualitative and quantitative analysis of non-polar compounds in (non-) infected *P. trichocarpa* roots and *P. cactorum* mycelium was conducted using a 6890 Series gas chromatograph (Agilent Technologies, Santa Clara, CA, USA) coupled to an Agilent 5973 quadrupole mass selective detector (interface temp, 270 °C; quadrupole temp, 150 °C; source temp, 230 °C; electron energy, 70 eV) or a flame ionization detector (FID) operated at 300 °C, respectively. The constituents of the hexane extracts were separated using a ZB5 column (Phenomenex, Aschaffenburg, Germany, 30 m × 0.25 mm × 0.25 μm) and He (MS) or H_2_ (FID) as carrier gas. The sample (1 μL) was injected without split at an initial oven temperature of 45 °C. The temperature was held for 2 min and then increased to 280 °C with a gradient of 6 °C min^−1^, and then further increased to 300 °C with a gradient of 60 °C min^−1^ and a hold of 2 min. Compounds were identified by comparing their retention times and mass spectra to those of authentic standards (Supplemental Tables S2 and S3), or to reference spectra in the Wiley and National Institute of Standards and Technology Libraries.

### 4.4. Methanol Extraction of Root Tissue and HPLC-UV, LC-MS/MS Analysis of Methanol Extracts

Metabolites were extracted from 40 mg fresh plant material by adding 1 mL 100% methanol (MeOH) containing 0.8 mg/mL phenyl-β-D-glucopyranoside (Sigma Aldrich, St. Louis, MO, USA) and 40 ng/mL D_6_-abscisic acid (D_6_-ABA) (Santa Cruz Biotechnology, Dallas, TX, USA) as internal standards. Samples were shaken for 30 sec in a paint shaker (Scandex, Büdelsdorf, Germany) and afterwards for 30 min at 200 rpm on a horizontal shaker (IKA Labortechnik, Staufen, Germany). After centrifugation, the supernatants were split for high performance liquid chromatography (HPLC)-UV and liquid chromatography-tandem mass spectrometry (LC-MS/MS) measurements.

Salicinoid analysis and quantification was performed by HPLC-UV (200 nm) as described previously in Böckler et al. [37] for the compounds salicin, salicortin, tremulacin, and homaloside D, and for 6′-*O*-benzoylsalicortin as described in Lackner et al. [38]. Chromatographic separation was achieved on an Agilent 1100 Series LC system (Agilent Technologies), using an EC 250/4.6 Nucleodur Sphinx column (RP 5 μm, Macherey-Nagel, Düren, Germany), with water and acetonitrile as mobile phases A and B, respectively. The mobile phase flow rate was 1 mL/min. The elution profile is listed in Supplemental Table S4 as gradient A. Salicinoids were quantified relative to the signal of the internal standard phenyl-β-D-glucopyranoside, by applying experimentally determined response factors [37,38].

The compounds salirepin, salicin-7-sulfate, and salirepin-7-sulfate were analyzed and quantified by LC-MS/MS as follows and as previously described in Lackus et al. [39]. Chromatographic separation was achieved using an Agilent 1260 infinity II LC system (Agilent Technologies) equipped with a Zorbax Eclipse XDB-C18 column (50 × 4.6 mm, 1.8 μm, Agilent Technologies), using aqueous formic acid (0.05% (*v/v*)) and acetonitrile as mobile phases A and B, respectively. The mobile phase flow rate was 1.1 mL/min. The elution profile is listed in Supplemental Table S4 as gradient B. The column temperature was maintained at 20 °C. The LC system was coupled to a QTRAP 6500^®^ tandem mass spectrometer (AB Sciex, Darmstadt, Germany) equipped with a turbospray ion source, operated in negative ionization mode. The ion spray voltage was maintained at −4500 eV and the turbo gas temperature was set at 700 °C. Nebulizing gas was set at 60 psi, curtain gas at 40 psi, heating gas at 60 psi and collision gas at medium level. Multiple reaction monitoring (MRM) was used to monitor analyte parent ion → product ion formation, and respective parameters are listed in Supplemental Table S5. Sulfated salicinoids and salirepin were quantified relative to the signal of the internal standard D_6_-ABA, by applying experimentally determined response factors [39]. Analyst 1.6.3 software (Applied Biosystems, Darmstadt, Germany) was used for data acquisition and processing.

### 4.5. RNA Extraction and Reverse Transcription

Total RNA was isolated from frozen and ground plant material using the InviTrap Spin Plant RNA Kit (Invitek, Berlin, Germany) according to the manufacturer’s instructions. RNA concentration was assessed using a spectrophotometer (NanoDrop 2000c, Thermo Fisher Scientific, Waltham, MA, USA). RNA was treated with DNaseI (Thermo Fisher Scientific) prior to cDNA synthesis. Single-stranded cDNA was prepared from 1 μg of DNase-treated RNA using SuperScript^TM^ III reverse transcriptase and oligo (dT_12_-_18_) primers (Invitrogen, Carlsbad, CA, USA).

### 4.6. Heterologous Expression of PtTPS5 and Enzyme Assays

*PtTPS5* was previously characterized by Irmisch et al. [12]. Based on its sequence deposited in GenBank with the accession number KF776503, *PtTPS5* was synthesized and cloned into pET100/D-TOPO vector (Thermo Fisher Scientific). The *Escherichia coli* strain BL21 Star™ (DE3) (Thermo Fisher Scientific) was used for heterologous expression. The culture was grown at 37 °C, induced at an OD_600_ = 0.6 with 1 mM IPTG, and subsequently placed at 18 °C and grown for another 20 h. The cells were collected by centrifugation and disrupted by a 4 × 20 s treatment with a sonicator (Bandelin UW2070, Berlin, Germany) in chilled extraction buffer (10 mM Tris-HCl (pH 7.5), 1 mM dithiothreitol, 10% (*v/v*) glycerol). Cell fragments were removed by centrifugation at 14,000 g and the supernatant was further processed via an Illustra NAP-5 gravity flow desalting column (GE Healthcare, Chicago, IL, USA) and eluted in extraction buffer.

Enzyme assays were performed in a Teflon-sealed, screw-capped 1 mL GC glass vial containing 50 μL of the heterologously expressed protein and 50 μL assay buffer containing 50 μM (*E,E*)-FPP substrate and 20 mM MgCl_2_. Assays were overlaid with 100 μL hexane and incubated for 60 min at 30 °C. One microliter of the hexane phase was injected into the GC-MS and the analysis was conducted using the same analytical parameters and equipment as described above for the analysis of poplar root hexane extracts. However, chromatographic separation was achieved with an initial oven temperature of 45 °C hold for 2 min, which was then increased to 180 °C with a gradient of 6 °C min^−1^, and then further increased to 300 °C with a gradient of 60 °C min^−1^ and a hold of 2 min.

### 4.7. RNA Sequencing and RT-qPCR Analysis

Total RNA was extracted from root material as described above, TruSeq RNA-compatible libraries were prepared, and PolyA enrichment was performed before sequencing eight transcriptomes of *P. trichocarpa*, four biological replicates (individual trees) each for the control and the oomycete treatments, on an IlluminaHiSeq 3000 sequencer (Max Planck Genome Centre, Cologne, Germany) with 45 Mio reads per library, 150 base pair, single end. Trimming of the obtained Illumina reads and mapping to the poplar gene model version 3.0 (https://phytozome.jgi.doe.gov/pz/portal.html) were performed with the program CLC Genomics Workbench (Qiagen Bioinformatics, Hilden, Germany) (mapping parameter: length fraction, 0.7; similarity fraction, 0.9; max number of hits, 25). Empirical analysis of digital gene expression (EDGE) implemented in the program CLC Genomics Workbench was used for gene expression analysis.

For RT-qPCR analysis, cDNA was prepared as described above and diluted 1:10 with water. Primers for gene expression analysis of *PtTPS5* and *Ypt1* were used as described in Irmisch et al. [12] and Schena et al. [18], respectively. *Ubiquitin (UBQ)*, *actin*, *elongation factor 1 alpha* (*EF1α*), *histone superfamily protein H3 (HIS)*, and *tubulin* (*TUB*) were tested as reference genes [40,41,42]. Primer sequences can be found in Supplemental Table S6. Comparison of ∆Cq values and the corresponding standard deviation revealed *HIS* as the most suitable reference gene for expression analysis in *P. trichocarpa* samples (Supplemental Table S7). Gene expression analysis was performed with an initial incubation at 95 °C for 3 min followed by 40 cycles of amplification (95 °C for 10 sec, 60 °C for 10 sec). For all measurements, plate reads were taken at the end of the extension step of each cycle and data for the melting curves were recorded at the end of cycling from 60 °C to 95 °C. All samples were run on the same PCR machine (Bio-Rad CFX Connect™ Real-Time PCR Detection System (Bio-Rad Laboratories, Hercules, CA, USA)) in an optical 96-well plate, using Brilliant^®^ III SYBR^®^ Green QPCR Master Mix (Stratagene, San Diego, CA, USA). Expression analysis was conducted for eight biological replicates in technical triplicates.

### 4.8. Compound Isolation and Structure Elucidation

The expression strain *E. coli* BL21 was transformed with the plasmid construct for PtTPS5 expression by electroporation. The cells were plated on LB agar containing ampicillin (100 mg mL^−1^) and incubated at 37 °C overnight. A single colony was selected from the plate and incubated in 10 mL of liquid LB medium at 37 °C overnight. The fresh culture was sequentially used to inoculate larger culture volumes (1 mL L^–1^, 8 L in total), followed by cultivation until an OD_600_ of 0.4–0.6 was reached. The cultures were cooled to 18 °C and IPTG solution (400 mM, 1 mL L^–1^) was added to induce protein expression. The cultures were grown overnight and then cells were harvested by centrifugation (3.600 × g, 40 min). The pelleted cells were resuspended in binding buffer (10 mL L^–1^ culture; 20 mM Na_2_HPO_4_, 500 mM NaCl, 20 mM imidazole, 1 mM MgCl_2_, pH = 7.4) and lysed by ultra-sonication (7 × 1 min). The supernatant obtained by centrifugation (11.000 × g, 10 min) contained the target protein for enzyme incubations.

The enzymatic assay was conducted in a total volume of 160 mL, containing 80 mL of enzyme preparation (with a protein concentration of 1.3 mg mL^–1^ as determined by Bradford assay), 80 mg (0.185 mmol) FPP trisammonium salt dissolved in 10 mL water, 304 mg (3.2 mmol) MgCl_2_ in 1.2 mL water (for a final concentration of 20 mM) and 68.8 mL incubation buffer (10 mM Tris-HCl, 1 mM dithiothreitol, 10% glycerol, pH 7.5). The incubation was performed at 28 °C overnight. The reaction mixture was extracted with pentane (3 × 150 mL), the extract was dried with MgSO_4_ and evaporated under reduced pressure to give 17 mg crude product. Purification by column chromatography on silica gel (pentane/ether = 4:1) and then HPLC (H_2_O/methanol = 25:75; 5:0 mL min^–1^; Smartline HPLC series; KNAUER Eurospher II 100-5 C_18_, 5 *μ*m, 250 × 8 mm) yielded (1*S*,5*S*,7*R*,10*R*)-guaia-4(15)-en-11-ol and (1*S*,7*R*,10*R*)-guaia-4-en-11-ol as colorless oils.

*(1S,5S,7R,10R)-Guaia-4(15)-en-11-ol***.** (**1**). Yield: 0.9 mg (0.004 mmol, 2%). TLC (pentane/ether = 4:1): *R*_f_ = 0.17. Optical rotation: [α]_D_^20^ = +47.8 (*c* 0.09, C_6_D_6_). HRMS (EI): *m*/*z* = 222.1978 (calc. for [C_15_H_26_O]^+^ 222.1978). GC (HP5-MS): *I* = 1660. MS (EI, 70 eV): *m*/*z* (%) = 222 (0.3), 204 (29), 189 (24), 175 (2), 161 (24), 149 (22), 133 (11), 121 (19), 107 (31), 91 (41), 81 (54), 67 (25), 59 (100), 53(13), 41 (35). IR (diamond ATR): 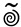/cm^–1^ = 2953 (m), 2923 (s), 2854 (m), 1714 (w), 1650 (w), 1456 (m), 1376 (m), 1260 (m), 1094 (s), 1020 (s), 873 (m), 800 (s).

*(1S,7R,10R)-Guaia-4-en-11-ol* (**2**)**.** Yield: 0.6 mg (0.003 mmol, 2%). TLC (pentane/ether = 4:1): *R*_f_ = 0.21. Optical rotation: [α]_D_^20^ = +21.7 (*c* 0.06, C_6_D_6_). HRMS (EI): *m*/*z* = 222.1975 (calc. for [C_15_H_26_O]^+^ 222.1978). GC (HP5-MS): *I* = 1661. MS (EI, 70 eV): *m*/*z* (%) = 222 (4), 204 (81), 189 (69), 175 (7), 161 (75), 147 (27), 133 (29), 119 (37), 105 (61), 91 (81), 79 (68), 67 (30), 59 (100), 51(5), 41 (55). IR (diamond ATR): 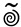/cm^–1^ = 2954 (s), 2923 (s), 2854 (s), 1723 (w), 1670 (w), 1459 (m), 1376 (m), 1260 (w), 1096 (w), 1025 (w), 800 (w).

Isotopic labelling experiments were performed to determine the absolute configurations of (1*S*,5*S*,7*R*,10*R*)-guaia-4(15)-en-11-ol and (1*S*,7*R*,10*R*)-guaia-4-en-11-ol. For the reactions with DMAPP (1 mg in 1 mL water) and (*Z*)-(4-^13^C,4-^2^H)IPP or (*E*)-(4-^13^C,4-^2^H)IPP (1 mg in 1 mL water), protein preparations of PtTPS5 (1 mL) and FPPS (1 mL, [22]), MgCl_2_ (19 mg, final concentration 20 mM) and incubation buffer (6 mL) were added. For the reactions with (*R*)-(1-^13^C,1-^2^H)IPP or (*S*)-(1-^13^C,1-^2^H)IPP (1 mg in 1 mL water), protein preparations of PtTPS5 (1 mL), FPPS (1 mL, [22]), IDI (1 mL, [21]), MgCl_2_ (19 mg, final concentration 20 mM) and incubation buffer (6 mL) were added. After incubation with shaking at 28 °C overnight, the reaction mixtures were extracted with C_6_D_6_, the extracts were dried with MgSO_4_ and analyzed by NMR and GC-MS.

### 4.9. Statistical Analysis

Throughout the manuscript, data are presented as means ± SE. Statistical analysis was performed with SigmaPlot 11.0 for Windows (Systat Software Inc., San Jose, CA, USA) and is described in the figure and table legends for the respective experiments. Whenever necessary, the data were log transformed to meet statistical assumptions such as normality and homogeneity of variances.

### 4.10. Accession Numbers

Raw reads of the RNAseq experiment were deposited in the NCBI Sequence Read Archive (SRA) under the BioProject accession PRJNA660564 ‘Oomycete-induced changes in the root transcriptome of poplar’.

## Figures and Tables

**Table 1:** Compounds detected in hexane extracts made from uninfected and *Phytophthora cactorum*-infected *Populus trichocarpa* roots. Means and SE in μg/g fresh weight are shown (*n* = 7–8).

Compound	Uninfected Roots	Infected Roots	t-Value/T-Value	*p*-Value
**Aromatic compounds**				
Benzylalcohol ^#^	0.22 ± 0.20	0.77 ± 0.20	48.00 (WR)	0.038 *
Salicylaldehyde ^#^	4.02 ± 1.00	4.93 ± 1.79	0.02 (ST)	0.988
2-Phenylethanol ^#^	0.11 ± 0.04	0.74 ± 0.23	39.00 (WR)	<0.001 ***
Benzyl salicylate	n.q.	n.q.	-	-
**Terpenes**				
Limonene ^#^	n.q	n.q	-	-
1,8-Cineole ^#^	n.q	n.q	-	-
α-Terpineol ^#^	0.10 ± 0.03	0.44 ± 0.05	5.26 (ST)	<0.001 ***
Elemol	0.01 ± 0.01	0.27 ± 0.09	40.50 (WR)	0.002 **
Guaia-4(15)-en-11-ol ^#^ + Guaia-4-en-11-ol ^#^	0.04 ± 0.04	4.31 ± 1.35	36.00 (WR)	<0.001 ***
**Fatty acids/aldehydes**				
(*E*)-4-Nonenal	n.q.	n.q.	-	-
Myristaldehyde	3.16 ± 0.34	8.53 ± 2.17	3.13 (ST)	0.007 **
Myristic acid ^#^	0.40 ± 0.08	6.13 ± 1.59	8.19 (ST)	<0.001 ***
Pentadecanoic acid ^#^	1.51 ± 0.30	4.49 ± 0.51	38.00 (WR)	<0.001 ***
Palmitic acid ^#^	24.53 ± 3.57	72.04 ± 6.13	36.00 (WR)	<0.001 ***
Oleic acid ^#^	5.04 ± 0.92	7.34 ± 1.09	1.51 (ST)	0.154
Stearic acid ^#^	1.65 ±0.19	5.29 ± 0.71	6.39 (ST)	<0.001 ***
**Others**				
1-Hexanol	0.14 ± 0.01	0.17 ± 0.01	12.00 (WR)	0.038 *
Unidentified compound	traces	1.64 ± 0.88	40.00 (WR)	0.002 **

Asterisks indicate statistical significance between uninfected roots and infected roots as assessed by Student’s *t*-test (ST) or Wilcoxon rank sum test (WR) (*, *p* ≤ 0.05; **, *p* ≤ 0.01; ***, *p* ≤ 0.001). n.q., not quantified due to trace amounts or incomplete separation. Compounds marked with # were identified using authentic standards.

**Table 2:** Salicinoids detected in methanol extracts made from uninfected and *Phytophthora cactorum*-infected *Populus trichocarpa* roots. Means and SE in μg/g fresh weight are shown (*n* = 7–8).

Compound	Uninfected Roots	Infected Roots	t-Value/T-Value	*p*-Value
Salicin	41.87 ± 16.03	77.22 ± 34.06	19.00 (WR)	0.336
Salicin-7-sulfate	2.08 ± 0.26	3.39 ± 0.39	2.52 (ST)	0.026 *
Salirepin	14.50 ± 2.39	20.62 ± 2.79	1.53 (ST)	0.151
Salirepin-7-sulfate	0.30 ± 0.03	0.42 ± 0.05	1.77 (ST)	0.1
Salicortin	368.31 ± 172.48	204.06 ± 70.32	2.25 (ST)	0.056
Tremulacin	3.16 ± 1.27	2.49 ± 1.98	0.28 (ST)	0.785
Homaloside D	16.77 ± 9.28	7.78 ± 3.04	33.00 (WR)	0.596

Asterisks indicate statistical significance between uninfected roots and infected roots as assessed by Student’s *t*-test (ST) or Wilcoxon rank sum test (WR) (*, *p* ≤ 0.05).

**Table 3:** NMR data of guaia-4(15)-en-11-ol and guaia-4-en-11-ol (isoguaiol B). NMR data were recorded on a 700 MHz spectrometer in C_6_D_6_ at 298 K. Coupling constants *J* are given in Hz and multiplicities are indicated by s = singlet, d = doublet, m = multiplet, br = broad.

C	Guaia-4(15)-en-11-ol (1)		Guaia-4-en-11-ol (2)	
	^13^C	^1^H	^13^C	^1^H
1	52.34 (CH)	1.18 (m)	55.60 (CH)	2.28 (m)
2	32.42 (CH_2_)	1.76 (m, H_α_) 1.00 (m, H_β_)	30.42 (CH_2_)	1.92 (m) 1.51 (m)
3	32.96 (CH_2_)	2.32 (ddm, *J* = 15.9, 7.8, H_β_) 2.19 (m, H_α_)	36.60 (CH_2_)	2.23 (m) 2.13 (m)
4	159.08 (C_q_)	–	131.74 (C_q_)	–
5	46.33 (CH)	2.15 (m)	138.62 (C_q_)	–
6	34.56 (CH_2_)	1.88 (ddd, *J* = 13.9, 9.2, 6.1, H_β_) 1.54 (ddd, *J* = 13.9, 11.3, 8.0, H_α_)	30.37 (CH_2_)	2.67 (d, *J* = 15.2) 2.00 (m)
7	49.56 (CH)	1.41 (m)	49.30 (CH)	1.36 (m)
8	26.71 (CH_2_)	1.74 (m, H_α_) 1.10 (m, H_β_)	31.51 (CH_2_)	1.90 (m) 1.00 (m)
9	40.29 (CH_2_)	1.77 (m, H_β_) 0.96 (m, H_α_)	40.26 (CH_2_)	1.78 (m) 1.10 (m)
10	42.32 (CH)	1.06 (m)	39.37 (CH)	1.33 (m)
11	73.26 (C_q_)	–	72.82 (C_q_)	–
12	27.75 (CH_3_)	1.03 (s)	26.89 (CH_3_)	1.03 (s)
13	25.78 (CH_3_)	1.01 (s)	26.66 (CH_3_)	1.02 (s)
14	21.58 (CH_3_)	0.87 (d, *J* = 6.5)	22.01 (CH_3_)	0.93 (d, *J* = 6.6)
15	104.24 (CH_2_)	5.00 (m, H*_E_*) 4.91 (br, H*_Z_*)	14.60 (CH_3_)	1.63 (br s)

## Data Availability

All data generated or analyzed during this study are included in the main text or supplement of this article. Raw sequences of the RNAseq experiment were deposited in the NCBI Sequence Read Archive under the BioProject accession PRJNA660564.

## Supplementary Materials

The following are available online, Figure S1: *Phytophthora cactorum* infection induces the accumulation of sesquiterpenes in *Populus trichocarpa* roots. (A) Pulverized root material was extracted with hexane and the extracts were analyzed using GC-MS. 1, elemol; 2, unidentified sesquiterpene alcohol 1; 3, unidentified sesquiterpene alcohol 2; 4, myristaldehyde. (B) *P. cactorum* infection was verified by RT-qPCR analysis of *Ypt1* (*Phytophthora*-specific Ras-related protein, Schena et al. [18]) gene expression. Means and SE are shown (*n* = 7–8). Asterisks indicate statistical significance as assed by Wilcoxon rank sum test (*** *p* < 0.001), (T = 36.00, *p* < 0.001). Figure S2: Representative GC-MS chromatograms of hexane extracts made from untreated *P. trichocarpa* roots, *Phytophthora cactorum*-treated *P. trichocarpa* roots, and *P. cactorum* mycelium. 1, 1-hexanol; 2, 1,8-cineole*; 3, benzyl alcohol*; 4, salicylaldehyde*; 5, (*E*)-4-nonenal; 6, 2-phenylethanol*; 7, α-terpineole*; 8, contamination (softener); 9, elemol; 10, (1*S*,5*S*,7*R*,10*R*)-guaia-4(15)-en-11-ol + (1*S*,7*R*,10*R*)-guaia-4-en-11-ol; 11, myristaldehyde*; 12, myristic acid*; 13, unidentified compound; 14, pentadecanoic acid*; 15, palmitic acid*; 16, oleic acid*; 17, stearic acid*; 18, unidentified compound; IS, internal standard (nonylacetate). Compounds marked by asterisks were identified using authentic standards. Figure S3: Determination of the absolute configuration of (1*S*,5*S*,7*R*,10*R*)-guaia-4(15)-en-11-ol by enantioselective deuteration. Partial HSQC spectra of A) unlabeled guaia-4(15)-en-11-ol (region for C2, C6 and C8), guaia-4(15)-en-11-ol obtained from B) (*R*)-(1-13C,1-2H)IPP and C) (*S*)-(1-13C,1-2H)IPP, D) unlabeled guaia-4(15)-en-11-ol (region for C3 and C9), and guaia-4(15)-en-11-ol obtained from E) (*E*)-(4-13C,4-2H)IPP and F) (*Z*)-(4-13C,4-2H)IPP. Taken together, these data establish the absolute configuration of (1*S*,5*S*,7*R*,10*R*)-guaia-4(15)-en-11-ol. Supplemental Table S1: Gene expression values (RPKM) and statistical parameters for all genes significantly upregulated (fold change > 5.0) upon *Phytophthora cactorum* infection in *Populus trichocarpa* roots. Supplemental Table S2: Compounds used as standards for GC-MS analysis. Supplemental Table S3: Experimentally determined Kovats retention indices. Supplemental Table S4: HPLC gradients used for separation and analysis of metabolites. Supplemental Table S5: Parameters used for LC-MS/MS analysis. Details of the HPLC gradient are given in Supplemental Table S4. CE, collision energy; DP, declustering potential; Q1, quadrupole 1; Q3, quadrupole 3. Supplemental Table S6: Primers used in this study. Supplemental Table S7: Ubiquitin (UBQ), *actin*, *elongation factor 1 alpha* (EF1α), *histone superfamily protein H3* (HIS), and *tubulin* (TUB) were tested as reference genes for RT-qPCR.

## Sample Availability

Samples of the compounds described in this study are available from the authors (J.S.D and T.G.K).
