# Supplementary material for: The Sesquiterpene Synthase PtTPS5 Produces (1S,5S,7R,10R)-Guaia-4(15)-en-11-ol and (1S,7R,10R)-Guaia-4-en-11-ol in Oomycete-Infected Poplar Roots
Source: Molecules. 2021 Jan 21;26(3):555. doi: 10.3390/molecules26030555 (PMC7866031; doi:10.3390/molecules26030555)
Supplement: Supplementary file 1 [file molecules-26-00555-s001.zip › 2021-01-15 supplemental figures_TGK.pptx]

## Slide 1
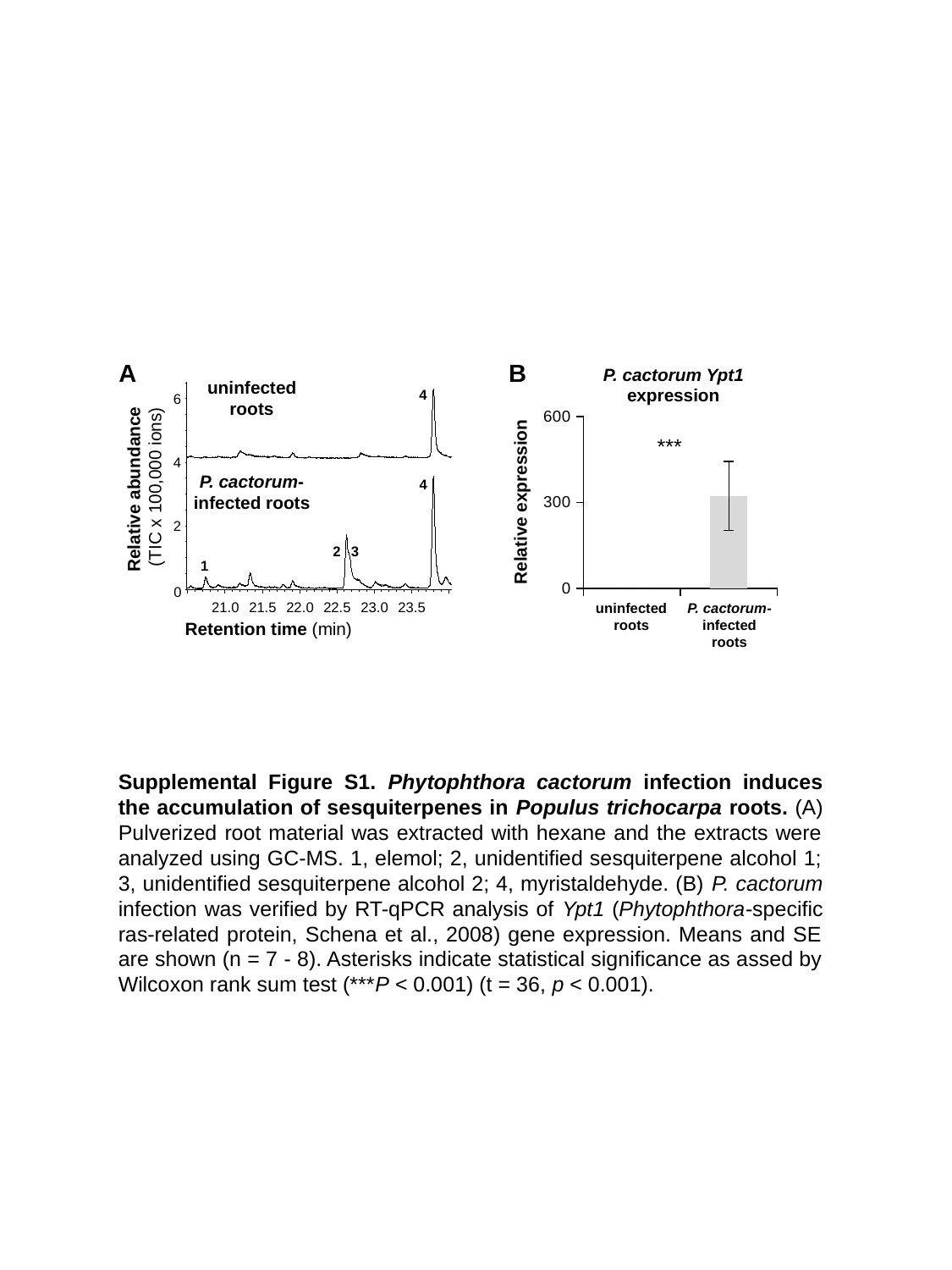

6
4
2
0
21.0
21.5
22.0
22.5
23.0
23.5
A
B
P. cactorum Ypt1 expression
### Chart
| Category | Average |
|---|---|
| Kontrolle | 0.0 |
| Infektion | 322.73274792342 |uninfected roots
4
***
Relative abundance
(TIC x 100,000 ions)
P. cactorum-infected roots
4
Relative expression
3
2
1
uninfected roots
P. cactorum-infected roots
Retention time (min)
Supplemental Figure S1. Phytophthora cactorum infection induces the accumulation of sesquiterpenes in Populus trichocarpa roots. (A) Pulverized root material was extracted with hexane and the extracts were analyzed using GC-MS. 1, elemol; 2, unidentified sesquiterpene alcohol 1; 3, unidentified sesquiterpene alcohol 2; 4, myristaldehyde. (B) P. cactorum infection was verified by RT-qPCR analysis of Ypt1 (Phytophthora-specific ras-related protein, Schena et al., 2008) gene expression. Means and SE are shown (n = 7 - 8). Asterisks indicate statistical significance as assed by Wilcoxon rank sum test (***P < 0.001) (t = 36, p < 0.001).

## Slide 2
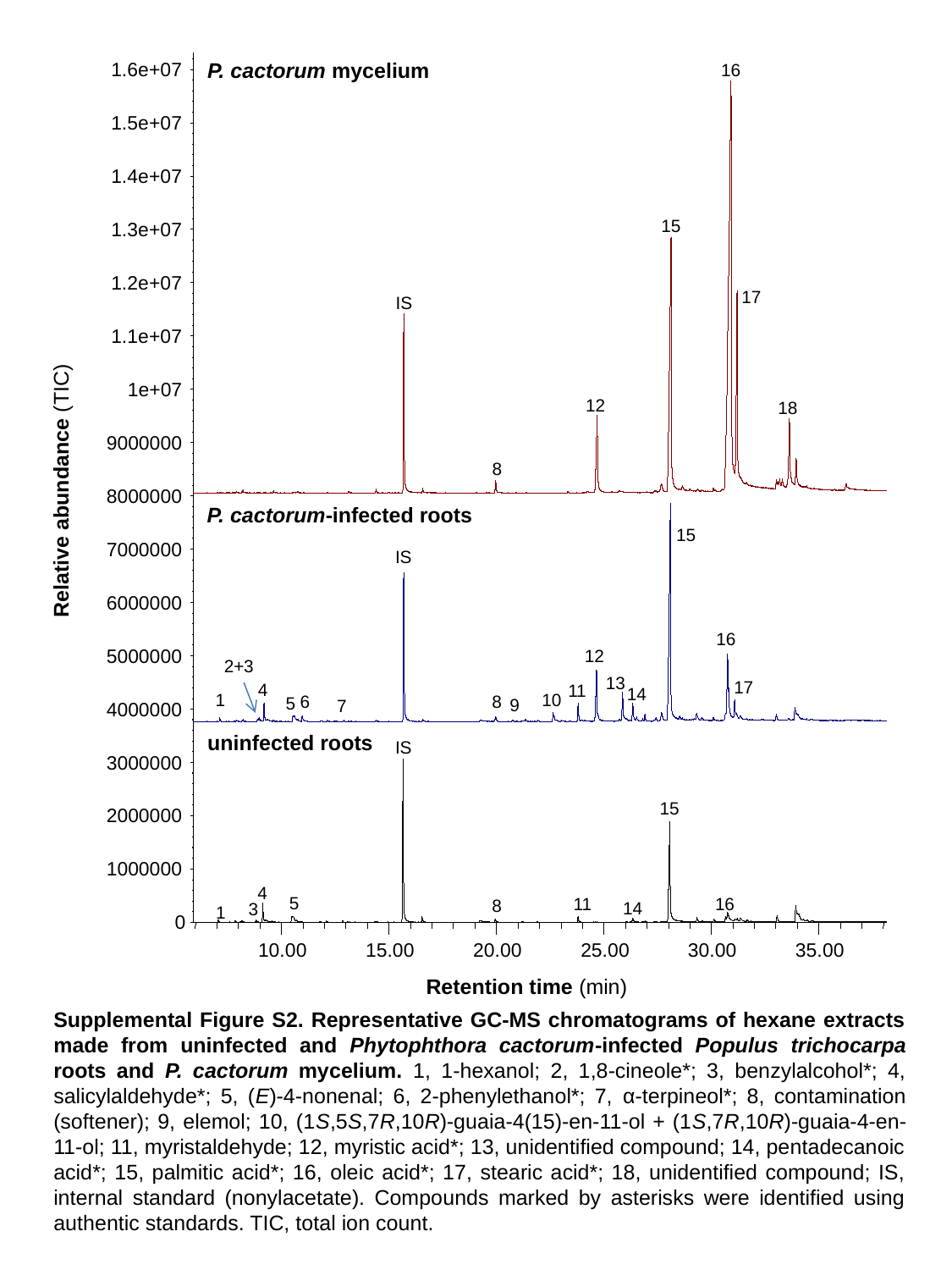

P. cactorum mycelium
16
 1.6e+07
 1.5e+07
 1.4e+07
15
 1.3e+07
 1.2e+07
17
IS
 1.1e+07
 1e+07
12
18
9000000
8
Relative abundance (TIC)
8000000
P. cactorum-infected roots
15
7000000
IS
6000000
16
12
5000000
2+3
13
17
4
11
14
1
10
6
8
5
9
7
4000000
uninfected roots
IS
3000000
15
2000000
1000000
4
5
11
16
8
14
3
1
0
10.00
15.00
20.00
25.00
30.00
35.00
Retention time (min)
Supplemental Figure S2. Representative GC-MS chromatograms of hexane extracts made from uninfected and Phytophthora cactorum-infected Populus trichocarpa roots and P. cactorum mycelium. 1, 1-hexanol; 2, 1,8-cineole*; 3, benzylalcohol*; 4, salicylaldehyde*; 5, (E)-4-nonenal; 6, 2-phenylethanol*; 7, α-terpineol*; 8, contamination (softener); 9, elemol; 10, (1S,5S,7R,10R)-guaia-4(15)-en-11-ol + (1S,7R,10R)-guaia-4-en-11-ol; 11, myristaldehyde; 12, myristic acid*; 13, unidentified compound; 14, pentadecanoic acid*; 15, palmitic acid*; 16, oleic acid*; 17, stearic acid*; 18, unidentified compound; IS, internal standard (nonylacetate). Compounds marked by asterisks were identified using authentic standards. TIC, total ion count.

## Slide 3
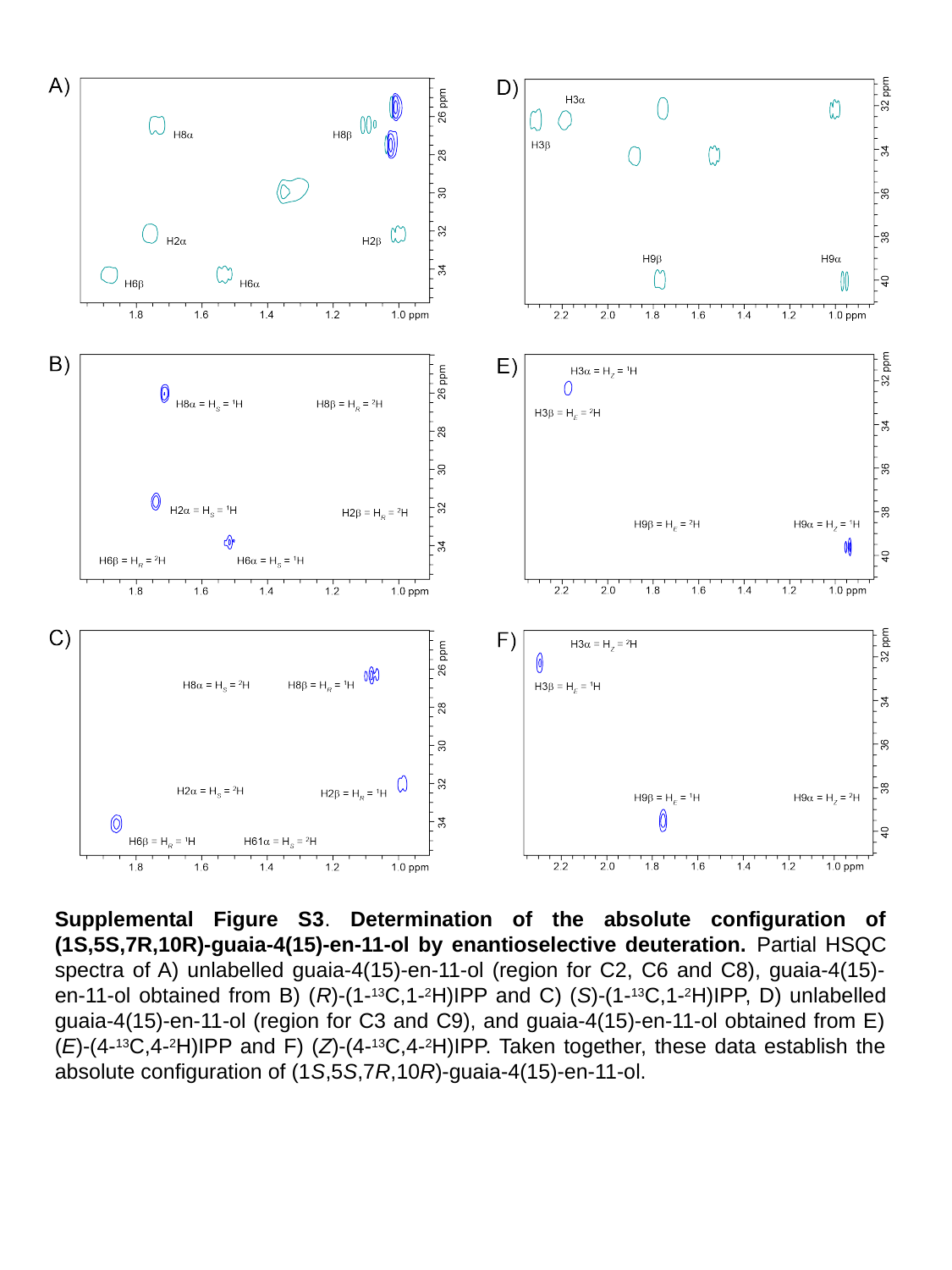

Supplemental Figure S3. Determination of the absolute configuration of (1S,5S,7R,10R)-guaia-4(15)-en-11-ol by enantioselective deuteration. Partial HSQC spectra of A) unlabelled guaia-4(15)-en-11-ol (region for C2, C6 and C8), guaia-4(15)-en-11-ol obtained from B) (R)-(1-13C,1-2H)IPP and C) (S)-(1-13C,1-2H)IPP, D) unlabelled guaia-4(15)-en-11-ol (region for C3 and C9), and guaia-4(15)-en-11-ol obtained from E) (E)-(4-13C,4-2H)IPP and F) (Z)-(4-13C,4-2H)IPP. Taken together, these data establish the absolute configuration of (1S,5S,7R,10R)-guaia-4(15)-en-11-ol.
